# Gluteus Maximus Transfer following Total Hip Arthroplasty Does Not Improve Abductor Moment: A Case-Control Gait Analysis Study of 15 Patients with Gluteus Medius Disruption

**DOI:** 10.3390/jcm11113172

**Published:** 2022-06-02

**Authors:** Roland Zügner, Natalie Hjelmberg, Ola Rolfson, Christer Strömberg, Tuuli Saari

**Affiliations:** 1 Department of Orthopaedics, Institute of Clinical Sciences, Sahlgrenska Academy, University of Gothenburg, 40530 Gothenburg, Sweden; natalie.hjelmberg.tiseth@vgregion.se (N.H.); ola.rolfson@vgregion.se (O.R.); jchst17@gmail.com (C.S.); tuuli.saari@vgregion.se (T.S.); 2 Department of Orthopaedics, Sahlgrenska University Hospital, 41345 Gothenburg, Sweden

**Keywords:** hip abductor strength, gait analysis, gluteus medius repair, hip arthroplasty

## Abstract

Gluteus maximus flap transfer (GMT) is a surgical technique used to improve gait kinematics and kinetics, as well as to reduce and ameliorate the functional outcome in patients with hip abductor deficiency following total hip arthroplasty (THA). The purpose of this observational study was to evaluate the gait pre- and postoperatively and examine whether GMT increases the abduction moment. Materials and Methods: A gait analysis based on reflective markers and force plates was performed in 15 patients who underwent GMT and were examined using an optical tracking system before and at a minimum of 13 months after the operation. The median follow-up time was 24 (13–60) months. The primary outcome was hip abduction moment (Nm/kg) during gait. The control group consisted of 15 female subjects without any gait pathology. Results: The mean adduction moment was significantly higher compared with controls before the operation (p = 0.02), but this did not apply to the abduction moment (p = 0.60). At the group level, the abduction moment did not improve postoperatively (p = 0.30). Only six of fifteen patients slightly improved their hip abduction moment postoperatively. However, speed (0.74 to 0.80 m/s) and cadence (94 to 105 steps/min) were improved (p < 0.03). Discussion: The results of this study showed no improvement in the hip abduction moment after GMT surgery. In our experience, abduction deficiency following primary THA is still a difficult and unsolved problem.

## 1. Introduction

Gluteus medius insufficiency caused by the destruction or disruption of the gluteus medius muscle is a known complication after hemi- and total hip arthroplasty (THA). There is a reported incidence of impaired gluteus medius function from mild to pronounced in 20% of all patients undergoing THA [1]. This insufficiency may cause limping and hip pain and patients often present with a positive Trendelenburg sign. Patients with abductor dysfunction run a higher risk of dislocation of the prosthesis [2]. As a complication of THA surgery, gluteus medius insufficiency is predominantly associated with the direct lateral approach. This approach releases the anterior insertions of the gluteus medius and minimus from the great trochanter of the proximal part of the femur. As a result of surgical trauma, the superior gluteal nerve may be injured, or the primary reattachment and healing of the muscle to the femur may fail [3,4]. Another cause could be the destruction of the femoral bone or the muscle itself due to the degenerative process [5,6].

Postoperative abductor insufficiency is usually investigated with PROM data or a static muscle test and, in the postoperative result assessment, subjective scores are sometimes filled in by biased assessors. No study that includes an objective dynamic gait analysis has been reported for this patient group with a pronounced impairment of gait and the need to use walking aids. A three-dimensional gait analysis pre- and postoperatively would facilitate an objective evaluation of the outcome of the surgical repair. Recordings of gait pattern with an optical tracking system (OTS) have commonly been used to study joint motion during gait in patients with osteoarthritis and after treatment with THA. There is a reported variability in gait performance in patients with THA and this variability is even greater in patients with hip osteoarthritis when compared with controls [7]. Compared with controls, reduced hip extension and range of motion of the hip, together with compensatory movements of the pelvis, knee and ankle, have been reported. Further, gait speed, stride length and peak hip abduction moment may be reduced [7,8,9,10,11,12,13].

There are several surgical repair techniques for an abductor tear, including direct transosseous repair, endoscopic repair, an Achilles tendon allograft and muscle transfer techniques. Relatively few cases have been reported and the success of these surgical repair techniques, in terms of reduced pain and improved function, is uncertain [1,6,14,15,16,17,18,19]. Further, no study that includes an objective gait analysis after surgical repair has been reported.

The surgical technique evaluated in our study is a modification of the surgical technique originally described by Whiteside [20]. In the study reported by Whiteside (2012), nine of ten patients displayed good strength when lifting the leg out sideways, no limp and a negative Trendelenburg sign at 16 months after surgery [20]. However, a gait analysis was not used in the study by Whiteside.

The purpose of this observational study was to objectively evaluate, using gait analysis, how the hip abduction moment and related walking parameters are influenced by gluteus maximus surgery and compare them with healthy controls.

## 2. Material and Methods

### 2.1. Subjects

**Figure 1:**
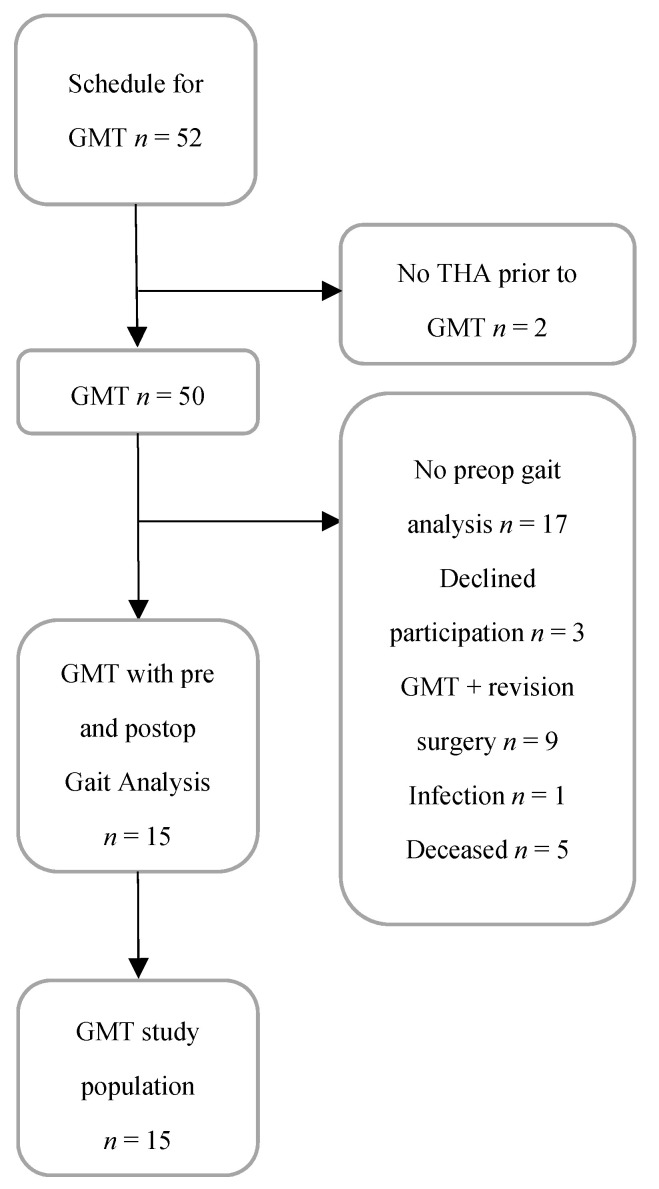
Flowchart of included patients.

Fifty-two patients with an abductor deficit due to the disruption of the gluteus medius were consecutively selected for surgery with GMT at Sahlgrenska University Hospital from December 2011 to April 2018. All patients with a pre- and postoperative gait analysis at the clinical follow-up were invited to participate in the study (Figure 1). An X-ray was performed preoperatively in order to exclude complications related to either the stem or the cup of the total hip arthroplasty. A gait analysis was used as a part of a clinical evaluation and, in this observational study, patients with both a pre- and postoperative gait analysis were included. Two of these patients had not undergone THA prior to the muscle transfer and were excluded. Five of the patients were deceased and three did not wish to participate. Furthermore, nine of the patients also underwent revision of either the acetabular or the femoral component of the total hip prosthesis or both components in the same session as the maximus flap transfer. Revision of an acetabular or femoral component may influence the outcome of the maximus flap transfer and these cases were excluded. Finally, one patient developed an infection postoperatively after GMT surgery and was also excluded. In this case, the maximus muscle was referred to as damaged during the flap transfer. Seventeen patients had no preoperative gait analysis, leading to 15 patients with a pre- and postoperative gait analysis with an optical tracking system (OTS). The surgery was performed within a median of 30 months (range 18–120) with an anterolateral incision with no surgical complications. All 15 of the included patients provided their consent to participate (Figure 1).

The GMT group consisted of women with a mean age of 69.5 years (range 47–82), weight of 78.2 kg (range 55–102), height of 1.65 m (range 1.6–1.8) and a BMI of 28.5 (range 22.6–36.1). Fifteen female individuals, with a mean age of 60 years (range 48–69) and a BMI of 25 (range 23.2–27.3), served as the control group. All of them declared that they had no other problems that would influence their walking ability.

The diagnosis of gluteus medius disruption was initially suspected due to persistent trochanteric pain and a positive Trendelenburg sign. All the patients were examined with ultrasound or magnetic resonance imaging (MRI) or both and the suspicion of a tear was verified with the exception of one patient. In this patient, the tear was confirmed at surgery, which meant that the patient was included in the study. Further, the tear was confirmed at surgery in all cases. A preoperative X-ray was performed in order to exclude complications related to either the stem or the cup of the total hip arthroplasty.

### 2.2. Surgery

The surgery was performed by three senior surgeons and the technique was consistent during the study period. Through a posterior incision, the gluteus maximus was split to approximately half the length of the muscle, in line with its fibres. The fascia just anterior to the gluteus maximus muscle was split in line with its fibres, extending from the iliac crest to well below the trochanter major. The incision created a V-shaped tongue at the end of the gluteus maximus muscle flap. The superior part of the gluteus maximus was used to replace the damaged part of the gluteus medius. The flap from the gluteus maximus was elevated. The greater trochanter was then prepared using a chisel or saw to allow the attachment of the anterior muscle flap. An attempt to mobilise the ruptured part of the gluteus medius was also made in order to be able to suture this muscle with the same type of sutures used for the maximus flap. The flap was attached to the greater trochanter with heavy non-absorbable sutures with the hip abducted 15 degrees. The vastus lateralis was split on the T-line and the distal, fibrous part of the maximus was placed under the muscle. The distal part of the flap was sutured under the vastus lateralis and the fascia lata was sutured side to side, in a V-Y fashion. Proximally, the edges were sutured towards the maximus muscle.

Postoperative care included partial weight-bearing with two crutches for six weeks. During this period, active hip abduction while standing was prohibited. Eight weeks after surgery, the patients were able to begin to perform abduction exercises while lying on the opposite side. Patients then began to gradually increase weight-bearing with only one crutch for another six weeks, as well as gradual abduction strengthening exercises.

### 2.3. Gait Analysis

A gait analysis was performed with a 12-camera (Qualisys^TM^, Gothenburg, Sweden) optical tracking system (OTS) with a sampling rate of 240 Hz, together with two force plates (Kistler^TM^, Winterthur, Switzerland) integrated in the floor, at one to two years after gluteus medius surgery. All the subjects wore underwear and walked barefoot over a walking distance of 10 m. In order to record kinematics and kinetics with the OTS, 15 spherical markers (ø 12 mm) were attached to the skin of the lower extremities and the pelvis, with double-adhesive tape, according to a skin marker model presented in detail elsewhere [13] which was reliability tested and validated [7,21,22]. One examiner (RZ) with more than 25 years’ experience of gait analysis attached all the markers. The reflective markers, according to the skin marker model, were attached to the proximal border of the sacrum, the anterior and superior iliac spine, the lateral knee joint line, the proximal border of the patella, the tibial tubercle, tuber calcanei, lateral malleolus and, finally, between the second and third metatarsals. In order to define the pelvis segment, a modified CODA pelvis was used. The two bilateral markers on the posterior superior iliac spine were replaced with one marker at the mid-point of the proximal border of the sacrum. According to recommendations by Bell et al., the hip-joint centres were defined in relation to the pelvis segment [8,23]. A static recording with the test subject standing in an upright position in the calibrated volume aligned to the global co-ordinate system was performed prior to the gait analysis in order to scale the subject’s anthropological measurements in relation to the marker positions. The test subjects were then asked to walk 5–10 times at a self-selected speed through the calibrated volume to familiarise themselves with the situation and then to perform six gait trials of which the approved trials for each test subject were selected for further evaluation. The mean of the approved trials for each test subject was used in the analysis to increase the reliability of the testing. A trial was excluded from the analysis if the patient missed stepping on the force plates correctly or due to other technical problems. The spatiotemporal variables that were collected were speed (m/s), step width (m), step length (m) and stance phase percentage (%) of the total gait cycle. The kinematic variables were degrees of hip extension and flexion, adduction and abduction. Kinetic variables collected in the frontal plane during the stance phase were moment (Nm/kg) and power (W/kg) in the joint. Prior to any calculations, the marker data obtained from the recordings were filtered using a Butterworth 4th filter with a cut-off frequency of 6 Hz. For calculations of spatiotemporal gait variables, kinematic and kinetic variables, Visual 3D™ software x64 Professional version 6.03.5 (C-Motion, Inc., Germantown, MD, USA) was used.

### 2.4. Radiographs

Radiographs were examined by one author (NH) using Mdesk software version 3.6.4 (RSA Biomedical Inc., Umeå, Sweden) in 11 of the 15 included patients. The offset was measured on the radiographs after the THA surgery and prior to the GMT. The offset was defined as the perpendicular distance from the teardrop through the femoral head centre of rotation to the axis of the femur [24]. This measurement was obtained bilaterally to calculate the difference in offset between the GTM hip and the contralateral native hip. This could not be measured in four patients due to a bilateral THA. In these four patients, adequate preoperative radiographs were missing. The leg length was measured from the apex of the lesser trochanter perpendicular to the trans-teardrop line (Table 1).

### 2.5. Statistics 

Non-parametric tests, the Wilcoxon signed rank test and the Mann–Whitney U test, were used, as some of the examined variables were not normally distributed. The results were analysed using SPSS Statistics version 25 (IBM SPSS New York, NY, USA), with the level of significance set at *p* < 0.05.

### 2.6. Ethics

The Regional Ethical Review Board in Gothenburg, Sweden (Dnr: 2851-18, 19 October 2018), approved the study. The implemented study only refers to patients who gave their consent to use data from the gait analysis, perform a review of medical records and assess X-ray images, together with a survey. Furthermore, all the patients received oral and written information about the study and were informed of their right to withdraw from the study at any time without any explanation. The patients signed a written consent form to participate in the study.

## 3. Results

### 3.1. Gait Analysis

The comparison between pre- and postoperative temporospatial gait variables revealed variables that increased. Walking speed (0.7 vs. 0.8 m/s) and cadence (94 vs. 105) increased due to an increase in the number of steps (p < 0.03) postoperatively. There was no difference regarding the kinematic or kinetic variables in the sagittal or frontal plane between preop and postop (Table 2).

**Figure 2:**
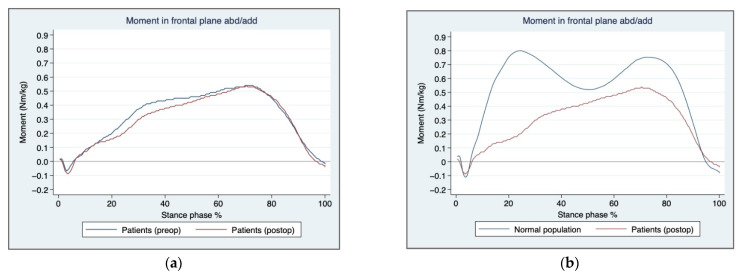
Hip abduction moment in the frontal plane: (**a**) Pre-and postoperative hip abduction moment during stance in patients, (**b**) Normal population and postoperative patients, hip abduction moment during stance.

In the GMT group, there was no statistically significant difference between the pre- and postoperative dynamic hip abduction moment, normalised to 100% during the stance phase in the frontal plane on the affected side (Figure 2a).

Furthermore, there was a lack of a dynamic component during the moment where the abduction muscles should contract twice during stance. This resulted in the absence of the specific M-shape graph which was observed in the healthy population (Figure 2b).

**Figure 3:**
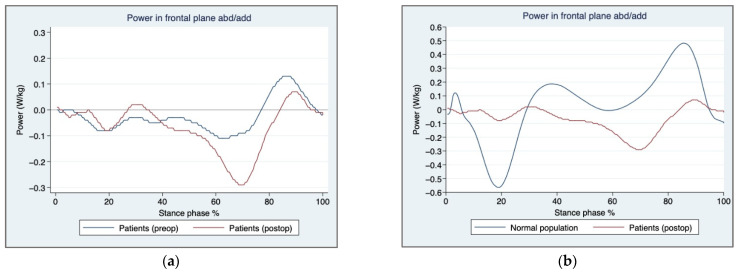
Hip power in the frontal plane: (**a**) Pre- and postoperative hip power absorption and generation in patients during stance, (**b**) Normal population and postoperative patients, hip power absorption and generation during stance.

During the stance phase, limited absorbed power was noted at the loading response and limited generated power at the terminal stance which was observed in the control group (Figure 3a,b).

Compared with controls, the patients generally had lower preoperative temporospatial gait parameters (p < 0.049). The patients also had less hip extension during stance (1.9° vs. −13.2°) and their hip flexion–extension range was smaller, at 30.4° compared with 41.2° (*p* < 0.001). Adduction was slightly higher, at 7.4° compared with 4.2°, but the difference was not significant, while abduction was lower, at −1.4° compared with −5.2° (*p* < 0.01). Changes in adduction moment were noted, of −0.6 Nm/kg and −0.3 Nm/kg, respectively (*p* = 0.02). No significant abduction and adduction–abduction moment range, 0.8 vs. 0.7 and 1.3 Nm/kg and 1.2 Nm/kg, respectively (*p* > 0.2), was observed (Table 2).

The postoperative comparison of the patients and the controls revealed lower values for speed, stride and stance (*p* < 0.001). Moreover, the kinematics of hip extension and the hip flexion-extension range were also lower, at 0.2° compared with −13.2° (*p* < 0.001) and 30.9°, compared with 41.2° (*p* = 0.003), respectively. Moreover, in the frontal plane, the kinematics for abduction were lower, at −1.4° compared with −5.2° (*p* = 0.01). Furthermore, the hip adduction moment differed significantly, at −0.6 vs. −0.3 Nm/kg (*p* = 0.01). No significant abduction moment and adduction–abduction moment range, of 0.7 vs. 0.7 and 1.2 vs. 1.2 Nm/kg, respectively (*p* > 0.30), was observed (Table 2).

### 3.2. Offset

The total offset showed a median difference of 2 mm (range −17–9) and a leg length discrepancy of 3 mm (range −5–16). Four of the patients underwent total hip arthroplasty surgery on the opposite side prior to the actual side, leading to radiographs that could not be measured (Table 2).

## 4. Discussion

The incidence of rupture or insufficiency of the gluteus medius muscle varies in the literature. In a study comprising 372 patients undergoing THA, the incidence was as low as 3.5% [25]. However, there are studies showing higher incidence. Whiteside et al. (2019) presented a study of 525 THA patients [1]. Almost 20% of the patients had mild or severe gluteus medius dysfunction. An incidence of abductor mechanism tear of 25% in older female patients and patients with a lower socioeconomic status was reported by Hendry [26].

In our study, 15 patients with GTM surgery after primary hip replacement underwent gait analysis with an OTS pre- and postoperatively, without any significant improvements in gait parameters except for speed and cadence. Compared with the controls, differences were noted, as has also been seen in other presented studies after THA [7,27,28]. Our results are in line with the study of Ruckenstuhl et al., who studied the results of gluteus maximus transfer [29]. In this study, 10 of 18 patients had an unchanged positive Trendelenburg test and the overall Harris Hip Score and abductor strength did not show any statistically significant improvement. Contrary to these results, Miozzari et al. reported that six of twelve patients did not have a positive Trendelenburg test or a limp after aponeurosis repair [30]. However, changes in abductor muscle strength were only slightly better, which is in line with our results.

Better clinical results after gluteus maximus transfer for the treatment of abductor insufficiency were reported by Christofipoulos et al. [31]. This study included 38 patients with variable hip history (native hips, after primary and secondary hip replacements) and demonstrated improvements in pain, the Harris Hip Score and abductor strength. However, the rating of abductor strength was based on how patients were able to abduct their hip against resistance. An objective gait analysis was not used. Moreover, 12 patients had a Trendelenburg limp postoperatively.

Defining walking ability or the severity of limping inclines a highly subjective assessment. Moreover, the Trendelenburg test, which is a static test of the hip muscles around the hip, is unreliable [32]. Furthermore, to test the function of the abductor muscles sideways on an examination board, both dynamically and statically against or without any resistance or weight, is not optimal or comparable for gait during single stance. Patients use two main techniques in gluteus medius insufficiency: lateral trunk flexion or lateral pelvic oblique, or a combination of the two. Both variants on the weak side occur during the stance phase. During this part of the gait cycle, the hip joint is stabilised by a number of hip muscles when the body is moving in forward propulsion. The use of an objective measurement instrument, such as gait analysis with synchronised cameras and force plates, appears to be the most reliable and feasible way to objectively evaluate the outcome of total hip prosthesis surgery and its muscular complications, such as gluteus medius insufficiency.

Moreover, Whiteside published two studies evaluating gluteus maximus transfer [1,20]. In the first study from 2012, and nine of 10 patients had good strength in abduction and no Trendelenburg limp after surgery. The authors explained the severe limp and weak abduction strength of one patient by old age, obesity and poor quality of the abductor muscles. In the later study, the function of the abductor muscles was examined with electrical stimulation before the decision to perform surgery with muscle transfer was made. GMT was only performed in the hip joints with a response from the abduction muscles to electrical stimulation. Further, the age of these 35 patients was not provided. In our study, all the patients scheduled for GMT underwent GMT without any examination of muscular contraction using electrical stimulation.

The preoperative gait analysis was performed in close connection to GMT surgery. At the group level, variable speed and cadence were improved after GMT surgery, but they remained significantly lower compared to the controls. This is in agreement with the findings presented in other gait analysis studies of THA patients [7,27]. However, according to kinematics in the sagittal plane, the patients remained in a flexed position during stance, which also affects the total range of motion in the sagittal plane. Together with less abduction in the frontal plane, this position, with more hip flexion and adduction during stance, appears to be more secure while loading the hip joint in the event of dysfunction in the gluteus medius muscle. The position makes it impossible for the gluteal muscles to stabilise the hip joint at initial contact and terminal stance. It also reflects the dysfunction or the absence of the abductor muscle function in the hip joint after surgery with GMT.

The shortening of the offset should always be considered as a cause of abductor muscle weakness after THA, but in this study we were unable to confirm it as a cause.

There are limitations to this study, such as the final number of patients included, the mean age and the BMI of the control group. Initially, 52 subjects with gluteus medius insufficiency were identified by carefully searching medical records and 37 were excluded for different reasons. The lack of information for four patients relating to the femoral offset and leg length might have affected the results. Three patients did not consent to participate without giving any reason, even though they had performed a pre- and postoperative gait analysis. Further, we wanted to identify a group of patients with as few confounding factors as possible, such as the replacement of any prosthetic component in connection with the GMT surgery, and this limited the selection. Three surgeons were involved, but they all used the same surgical technique described above and we do not feel this had a decisive effect on the final result.

One way to compare groups using a gait analysis system is to compare maximum values during the stance phase. If patients with gluteus medius insufficiency lean passively against the structures around the hip joint, an almost comparable maximum moment value is generated during the stance phase. No dynamic muscular moment at initial contact and at terminal stance, related to the specific M-shape (Figure 2b), is generated. This might be the reason as to why the abduction moment values failed to reach significance and need to be addressed in future studies.

Moreover, there is a known relative displacement between skin and skeleton which has been presented in several studies. Further, our group compared radiostereometric analysis and OTS [22]. In this study, tantalum markers of 1 mm were inserted in the skeleton of the pelvis and femur, together with the skin-attached reflective markers used in OTS. A dynamic squat was performed by the patient during synchronised registration by both systems. The results of this study were more advantageous for movements in the sagittal plane compared with the frontal plane, but they were still acceptable. Moreover, we also conducted a reliability study comparing patients with unilateral hip osteoarthritis, a unilateral hip prosthesis and healthy controls using OTS [7]. This study revealed that patients with THA did not report having the same walking ability as healthy individuals, despite the fact that they experienced less pain and greater satisfaction with the THA, illustrating results that are significant.

## 5. Conclusions

In conclusion, the objective gait analysis in this study shows that GMT surgery unfortunately failed to improve the abduction moment, which was severely impaired compared with healthy controls. Abduction deficiency following primary THR is still a difficult and unsolved problem and new surgical strategies and studies which focus on objective gait analysis are required.

## Figures and Tables

**Table 1:** The total postoperative offset and leg length of 11 patients included in the study. The calculated difference between the offset and the leg length measurement of the gluteus maximus transfer (GMT) side and the contralateral side (REF) is presented in millimetres (mm).

Patient Number	Offset GMT	Offset REF	Diff GMT vs. REF.	Leg Length GMT	Leg Length REF	Diff GMT vs. REF
1	76	74	2	46	43	3
2	54	71	−17	49	42	7
3	74	72	2	54	54	0
4	64	60	4	46	30	16
5	73	80	−7	39	44	−5
6	72	63	9	46	44	2
7	70	72	−2	47	41	6
8	63	70	−7	48	35	13
9	68	75	−7	50	43	7
10	73	76	−3	41	44	−3
11	68	62	6	48	47	1
Min	54	60	−17	39	30	−5
Max	76	80	9	54	54	16
Median	70	72	−2	47	43	3

**Table 2:** Gait parameters in 15 controls and 15 patients undergoing gluteus maximus transfer pre- and postoperatively at the two-year follow-up.

	GMT-Pre *n* = 15		GMT-Post *n* = 15		Controls *n* = 15		GMT-Pre vs. GMT-Post	Comparison with Controls	
								GMT-Pre	GMT-Post
	Mean	95% CI	Mean	95% CI	Mean	95% CI	*p*-Value ^#^	*p*-Value *	*p*-Value *
Speed (m/s)	0.74	0.62–0.86	0.80	0.68–0.91	1.19	1.1–1.3	0.029	<0.001	<0.001
Stride (m)	0.89	0.76–1.02	0.86	0.72–0.99	1.3	1.2–1.4	0.49	<0.001	<0.001
Cadence (steps/min)	94.2	86.1–102.2	105.3	92.6–118.0	103.6	95.1–112.1	0.015	0.049	0.76
Stance (%)	65.3	63.6–67.0	65.7	63.8–67.5	61.5	60.3–62.7	0.65	<0.001	<0.001
Hip extension°	1.89	−3.5–7.2	0.17	−4.9–5.2	−13.2	−16.4–(−9.9)	0.46	<0.001	<0.001
Hip flexion°	32.2	28.5–35.9	31.1	28.0–34.2	28.0	24.8–31.1	0.39	0.10	0.14
Hip ext-flex range°	30.4	25.3–35.5	30.9	26.1–35.7	41.2	38.4–43.9	0.53	<0.001	0.003
Hip adduction°	7.4	4.8–10.0	7.3	3.8–10.7	4.2	2.3–6.2	0.91	0.08	0.19
Hip abduction°	−1.4	−4.0–1.2	−1.4	−4.6–1.8	−5.2	−7.8–(−2.6)	0.86	0.01	0.01
Hip add-abd range°	8.8	6.6–11.0	8.6	6.3–10.9	10.5	8.5–12.5	0.33	0.18	0.18
Hip adduction moment Nm/kg	−0.56	−0.70–(−0.42)	−0.56	−0.66–(−0.46)	−0.32	−0.48–(−0.17)	0.28	0.02	0.012
Hip abduction moment Nm/kg	0.78	0.58–0.95	0.67	0.49–0.85	0.71	0.41–1.02	0.30	0.60	0.30
Hip add-abd moment range Nm/kg	1.34	1.13–1.55	1.25	1.01–1.48	1.20	1.04–1.30	0.70	0.18	0.49

^#^ Wilcoxon signed rank test; * Mann–Whitney U test; GMT = gluteus maximus transfer; CI = confidence interval, ° = degree.

## Data Availability

All data are available on request.
